# Long-Term Evaluation in Aesthetic Rhinoplasty in an Academic Referral Center

**DOI:** 10.1590/S1808-86942010000400006

**Published:** 2015-10-19

**Authors:** Gabriel Bijos Faidiga, Lucas Rodrigues Carenzi, Camila Carrara Yassuda, Flavia Silveira, Tassiana do Lago, Marcelo Gonçalves Junqueira Leite, Wilma Terezinha Anselmo-Lima

**Affiliations:** a MD - FMRP-USP, Third Year Resident in ENT - Department of Ophthalmology, Otorhinolaryngology and Head and Neck Surgery - Ribeirão Preto Medical School - University of São Paulo (FMRP-USP); b MD - FMRP-USP, Third Year Resident in ENT - Department of Ophthalmology, Otorhinolaryngology and Head and Neck Surgery - Ribeirão Preto Medical School - University of São Paulo; c MD - FMRP-USP, Third Year Resident in ENT - Department of Ophthalmology, Otorhinolaryngology and Head and Neck Surgery - Ribeirão Preto Medical School - University of São Paulo; d MD - FMRP-USP, Third Year Resident in ENT - Department of Ophthalmology, Otorhinolaryngology and Head and Neck Surgery - Ribeirão Preto Medical School - University of São Paulo; e MD - FMRP-USP, Third Year Resident in ENT - Department of Ophthalmology, Otorhinolaryngology and Head and Neck Surgery - Ribeirão Preto Medical School - University of São Paulo; f MD - FMRP-USP, Assistant Physician - Department of Ophthalmology, Otorhinolaryngology and Head and Neck Surgery - Ribeirão Preto Medical School - University of São Paulo; g Associate Professor - University of São Paulo (USP); Head of the Otorhinolaryngology Division - Department of Ophthalmology, Otorhinolaryngology and Head and Neck Surgery -Ribeirão Preto Medical School - University of São Paulo

**Keywords:** esthetics, surgery, plastic, rhinoplasty

## Abstract

Plastic surgery is based on improving esthetic for the patient. In most services, the surgery outcome is evaluated in a subjective manner.

**Aim:**

to objectively assess the degree of patient satisfaction one year after rhinoplasty using the Rhinoplasty Outcome Evaluation questionnaire at a referral academic center.

**Materials and Methods:**

69 patients operated in the otorhinolaryngology service were selected. The patients were operated upon by third year residents during the period from January to December 2007 and answered the questionnaire translated by the authors of this study.

**Results:**

we obtained a mean value of 73.25% of satisfaction for primary rhinoplasty and a mean value of 72.02% of satisfaction for secondary rhinoplasty.

**Conclusion:**

the level of satisfaction presented by the patients was considered to be very good.

## INTRODUCTION

In Brazil, results-driven medicine is on the rise, as are cosmetic surgeries. Rhinoplasty can be broken down into cosmetic rhinoplasty, functional or post-traumatic^1^. Regardless of demand, in our Otorhinolaryngology Department this nasal surgery aims at functional and cosmetic correction. Cosmetic surgery is discussed with the patient as to expectations, wish and surgical objectives; functional surgery aims at maintaining or improving nasal breathing.

In recent decades there has been a growing interest in assessing surgery results in many medical subspecialties. In otorhinolaryngology this focus is mainly geared to specific disorders or oncological diseases^2^, ^3^, ^4^, ^5^, ^6^. In regards of facial plastic surgery, despite patient satisfaction being highly relevant in this procedure, there are very few studies about it. There are more studies assessing patient satisfaction after blepharoplasty^7^ and otoplasty^8^ instead of rhinoplasty.

The result of any surgical procedure can be defined in many different ways, both in quantitative as in qualitative terms. Differently from oncology, metrics such as the morbidity or mortality associated with a given procedure mean very little in facial plastic surgery, where most of the procedures are elective and cosmetic. Usually, in this field one way of assessing is the subjective analysis made by both the patient and the surgeon. Thus, there is an information gap - through which, one could compare different techniques and different surgeons.

Patient satisfaction is the principal means used to measure the results of facial cosmetic surgeries. It is meaningless to have the surgeon pleased with the procedure, but the patient is unhappy with it. In such a case, the procedure cannot be considered a successful one.

Surgeons are always interested in the result of a surgical procedure. Efficacy can be measured in clinical trials^9^. Considering surgical results, it is necessary to employ a method to measure and assess the level of patient satisfaction, quality of life and organ function.

In the year 2000, Alsarraf^10^ proposed four questionnaires with the goal of assessing the results obtained from facial cosmetic surgeries, transforming a subjective data from a patient into a quantitative one. They are: ROE (Rhinoplasty Outcome Evaluation), FOE (Facelift Outcome Evaluation), BOE (Blepharoplasty Outcome Evaluation) and the SROE (Skin Rejuvenation Outcome Evaluation).

The ROE has 6 questions, which can be answered by the patient during pre-op and post-op assessments:

1 Do you like the looks of your nose?;
2 Can you breathe through your nose?;
3 Do you think your friends and the ones dear to you like your nose?;
4 Do you think the looks of your nose limit your social and professional activities?;
5 Is your nose closer to perfection?;
6 Would you like to surgically correct your nose's function or looks?

Each one of the questionnaire questions has five answers ranked from 0 to 4, whereas 0 means the most negative answer and 4 the most positive one. In order to reach the final result of the scale, one must add up the scores from each questionnaire and divide it by 24. The result is then multiplied by 100. We then have an interval value from 0 to 100, where 0 represents the unhappiest patient and 100 the most pleased of them.

## OBJECTIVE

To assess the degree of patient satisfaction one year after the cosmetic rhinoplasty in a medical residency facility, using the ROE questionnaire.

## MATERIALS AND METHODS

From January of 2007 through December of 2007, all the patients submitted to rhinoplasty by third year medical residents in our department of otorhinolaryngology were asked, through a letter, to come to our ward at a given date and time in order to answer an ROA questionnaire. We took off the study those patients who, besides rhinoplasty were also submitted to other associated procedures (septoplasty, endoscopic sinus surgery, turbinectomy, etc.), as well as those operated by professors and assistant physicians.

To each patient who came, we presented the study goals, risks and benefits, and we gave the patient a Free and Informed Consent Form to sign. From the total of 127 patients invited by letter, 69 came and accepted to participate in the study. These 69 patients received the ROE questionnaire translated by the authors of the study to fill out and return to the investigators. Data concerning the type of procedure performed was carefully checked from the patients' surgery records.

The data was processed in a Microsoft Excel spreadsheet, where we calculated the mean and standard deviation values.

The study was approved by the Ethics in Research with Human Beings Committee of the hospital, under protocol # 1461/2009.

## RESULTS

Of the 69 patients selected, 54 were females and 15 were males. The mean age was 28.9±8.5 years. Of these, 62 were submitted to primary rhinoplasty and 7 to secondary rhinoplasty. All the patient had at least one year of post-op time, varying between 1 and 4 months and 2 and 5 months.

The answers from the patients were individually analyzed and, following that, we obtained a mean value. Concerning the degree of satisfaction with the surgery as far as cosmetics and respiratory functions were concerned, on the ROE scale the mean value was 73.25% of satisfaction, varying between 25 and 100%, for primary rhinoplasty and 72.02% varying between 45.83 and 91.67% for secondary rhinoplasty. (Table 1, Table 2)

**Table 1:** Patients submitted to primary rhinoplasty.

Patient	Age	Question 1	Question 2	Question 3	Question 4	Question 5	Question 6	SCORE ROE
1	20	2	1	3	4	1	1	50,00%
2	24	1	2	0	2	1	0	25,00%
3	37	3	4	3	4	3	4	87,50%
4	27	4	3	4	4	4	4	95,83%
5	38	3	3	4	4	3	1	75,00%
6	48	3	2	3	2	3	2	62,50%
7	28	1	2	2	4	2	0	45,83%
8	24	4	4	4	4	4	4	100,00%
9	39	4	3	3	4	1	4	79,17%
10	28	1	1	2	4	2	0	41,67%
11	39	4	4	4	4	3	4	95,83%
12	36	3	3	4	4	3	2	79,17%
13	36	3	4	3	4	2	1	70,83%
14	19	4	3	4	4	3	4	91,67%
15	19	3	4	3	4	3	1	75,00%
16	36	3	3	3	4	3	4	83,33%
17	44	4	3	4	4	4	4	95,83%
18	24	1	4	1	2	0	0	33,33%
19	42	2	3	1	4	3	0	54,17%
20	23	4	3	4	1	3	4	79,17%
21	21	2	2	2	4	2	4	66,67%
22	24	3	1	4	4	3	0	62,50%
23	27	2	4	2	4	2	2	66,67%
24	24	4	3	4	4	4	4	95,83%
25	16	4	4	4	4	3	4	95,83%
26	31	3	4	4	4	2	4	87,50%
27	26	4	3	4	4	4	4	95,83%
28	27	2	2	3	3	3	1	58,33%
29	27	2	4	2	4	2	3	70,83%
30	21	4	2	4	4	4	3	87,50%
31	41	3	4	3	4	3	4	87,50%
32	25	3	2	3	4	3	1	66,67%
33	47	4	3	4	2	4	4	87,50%
34	27	3	4	4	4	3	4	91,67%
35	28	3	2	2	4	2	0	54,17%
36	19	3	2	3	4	3	4	79,17%
37	24	2	4	2	4	2	1	62,50%
38	24	1	0	1	3	1	0	25,00%
39	38	3	2	4	4	3	4	83,33%
40	42	4	2	3	4	4	4	87,50%
41	22	1	1	2	2	2	1	37,50%
42	29	3	3	3	4	3	4	83,33%
43	32	2	4	0	4	2	0	50,00%
44	26	2	4	2	4	2	2	66,67%
45	28	3	3	3	4	3	3	79,17%
46	26	3	3	4	3	3	4	83,33%
47	30	2	2	3	4	2	0	54,17%
48	25	4	4	4	4	4	4	100,00%
49	22	4	3	4	2	3	4	83,33%
50	25	2	4	2	2	2	0	50,00%
51	22	2	3	4	4	1	1	62,50%
52	22	3	3	4	4	3	4	87,50%
53	30	4	4	4	4	4	4	100,00%
54	27	4	1	4	4	4	0	70,83%
55	19	3	3	3	3	2	4	75,00%
56	20	4	3	4	4	3	4	91,67%
57	28	1	2	1	4	2	0	41,67%
58	18	4	2	4	4	3	4	87,50%
59	23	2	2	3	4	0	4	62,50%
60	19	4	4	3	4	3	2	83,33%
61	20	4	3	3	4	4	4	91,67%
62	40	2	4	3	2	2	3	66,67%

**Table 2:** Patients submitted to secondary rhinoplasty.

Patient	Age	Question 1	Question 2	Question 3	Question 4	Question 5	Question 6	SCORE ROE
63	48	3	3	3	3	3	3	75,00%
64	53	3	2	3	4	3	3	75,00%
65	35	4	2	4	4	4	4	91,67%
66	22	4	2	4	4	4	3	87,50%
67	29	1	2	2	4	2	0	45,83%
68	28	3	3	4	2	3	1	66,67%
69	36	3	2	3	4	2	1	62,50%

## DISCUSSION

With the ROE questionnaire we can quantify the result from the surgical treatment proposed, assessing quality of life, respiratory function and the cosmetic result desired by the patient submitted to rhinoplasty. It is also possible to assess the improvement or worsening of the patients' complaints by employing the questionnaire before and after surgery.

Often times it is difficult for the surgeon to judge the result from the rhinoplasty, or even when the surgeon considers the surgery's result as being short of what was expected by the patient. Nonetheless, using the ROE questionnaire we can have an accurate idea of the patient's satisfaction.

In an attempt to compare our results to those described in the literature from reference academic center we find only those from Hellings & Trenité (2007)^11^. Using a questionnaire with patients who had already been operated but were unhappy with the first procedure, they obtained a mean value of 42.8 ±2.7. After the second procedure, this value went up to 58.8 ±2.8. Guyuron & Bokhari (1996)^12^, used a simpler method of questions and answers concerning the degree of patient satisfaction after the first rhinoplasty, they found 87% of satisfaction among women and 62% among men. Although our results were equivalent in percentages concerning the level of satisfaction presented by the patients, the methodology used was not the same.

By analyzing the data (concerning the level of satisfaction) obtained in this study (73.25%±19.42 for primary rhinoplasty and 72.02%±15.54 for secondary rhinoplasty), it is worth to stress that even in a clinic in which the patients are submitted to rhinoplasty - considered the procedure with the lowest level of patient satisfaction when compared to other types of cosmetic surgery,^8^^,^^10^ the level of satisfaction is very good. It is worth bearing in mind that the surgeries were performed by third year residents, in other words, trainees.

We believe there is a bias in our study which must be taken into account when assessing the methodology employed. It happened with the letter sent to the patients explaining the reason for the visit, we believe that many of the patients who did not come for this evaluation were pleased with the outcome and therefore were not interested in this study. In the papers already published, the ROE questionnaire was sent to people by mail.

## CONCLUSION

Despite being trainees doing rhinoplasty in a teaching setting, always under guidance, patient satisfaction was very good. We believe that the ROE questionnaire was a useful method and easy to employ in order to assess the postoperative results of rhinoplasty.
